# Impact of UV-C Irradiation on Bacterial Disinfection in a Drinking Water Purification System

**DOI:** 10.4014/jmb.2211.11027

**Published:** 2022-11-30

**Authors:** Hyun-Joong Kim, Hee-Won Yoon, Min-A Lee, Young-Hoon Kim, Chang Joo Lee

**Affiliations:** 1 Department of Food Engineering, Mokpo National University, Muan, 58554, Republic of Korea; 2 Department of Food Science and Biotechnology, Wonkwang University, Iksan, Jeonbuk 54538, Republic of Korea

**Keywords:** Ultraviolet-C, drinking water, *Shigella flexneri*, *Listeria monocytogenes*, flow cytometry, fluorescence microscopy

## Abstract

The supply of microbiological risk-free water is essential to keep food safety and public hygiene. And removal, inactivation, and destruction of microorganisms in drinking water are key for ensuring safety in the food industry. Ultraviolet-C (UV-C) irradiation is an attractive method for efficient disinfection of water without generating toxicity and adversely affecting human health. In this study, the disinfection efficiencies of UV-C irradiation on *Shigella flexneri* (Gram negative) and *Listeria monocytogenes* (Gram positive) at various concentrations in drinking water were evaluated using a water purifier. Their morphological and physiological characteristics after UV-C irradiation were observed using fluorescence microscopy and flow cytometry combined with live/dead staining. UV-C irradiation (254 nm wavelength, irradiation dose: 40 mJ/cm^2^) at a water flow velocity of 3.4 L/min showed disinfection ability on both bacteria up to 10^8^ CFU/4 L. And flow cytometric analysis showed different physiological shift between *S. flexneri* and *L. monocytogenes* after UV-C irradiation, but no significant shift of morphology in both bacteria. In addition, each bacterium revealed different characteristics with time-course observation after UV-C irradiation: *L. monocytogenes* dramatically changed its physiological feature and seemed to reach maximum damage at 4 h and then recovered, whereas *S. flexneri* seemed to gradually die over time. This study revealed that UV-C irradiation of water purifiers is effective in disinfecting microbial contaminants in drinking water and provides basic information on bacterial features/responses after UV-C irradiation.

## Introduction

The human body is composed of more than 60% water, which makes up the largest proportion of the organs [1]. Humans consume ~ 2 L of water per day via drinking water and fresh and processed foods, which plays an important role in metabolic processes within the body [2]. In addition, water is also used for washing fresh and processed foods. Water-borne diseases have been a concern to humans ever since their cause was discovered, and the most appropriate treatment process adopted is microbial disinfection [3]. Inadequate access to safe water contributes to nearly 1.7 billion episodes of diarrhea per year worldwide [4]. In addition to having water for drinking and washing fresh and processed foods, it is important to have access to water for hygiene purposes, which is crucial for diarrhea episodes [5]. The microbial safety of water is an important factor in determining food and public hygiene [6]. However, water is inevitably contaminated during water intake or storage by the microorganisms present in the environment. Therefore, the removal, inactivation, and destruction of microorganisms present in water are important to promote public hygiene and microbial stability of food [7]. Drinking water is traditionally consumed after heat sterilization. Microorganisms are killed or inactivated by physical and chemical changes that occur at high temperatures. Water contaminated with microorganisms can be effectively sterilized and inactivated by heat treatment. Heat sterilization treatment of drinking water and water for food production is economical, considering the amount of energy required for heating and cooling time but is not suitable in terms of production efficiency. Among the non-thermal physical methods, ultraviolet (UV) light is considered a cost-effective and easily implementable disinfection method for drinking water. In addition, ultraviolet irradiation sterilization can efficiently control contaminants without generating toxic substances, and chemical and microbial safety can be guaranteed simultaneously [8-10]. Interest in UV disinfection processes has increased sharply in the drinking water industry since researchers in the late 1990s demonstrated that even very low doses of UV light could effectively inactivate *Cryptosporidium* [11-13]. Ultraviolet is electromagnetic radiation in the wavelength range of 10–400 nm, depending on the wavelength, UV-A (wavelength: λ=315–400 nm), UV-B (λ=280–315 nm), UV-C (λ=100–280 nm), and EUV (extreme UV, λ=10–121 nm) (Jeon and Ha, 2018). UV disinfection primarily occurs because of the germicidal action of UV-C light in the range of 200–280 nm on microorganisms [14]. Thus, UV-C is called germicidal radiation and has maximum effect at a wavelength of 260 to 265 nm when absorbed by DNA and protein [15]. UV irradiation directly destroys the DNA of microorganisms or acts on pyrimidines (cytosine, C; thymine, T) in DNA, and the double helix structure of DNA where pyrimidine is located is disrupted, finally killing bacteria [16, 17]. Therefore, UV irradiation causes microbial DNA to lose its physical and chemical structural properties, inhibits the production of metabolites for microbial growth, and prevents DNA replication, leading to death [18]. In general, UV-C is used to sterilize water and food surfaces, whereas UV-A and UV-B are effective for sterilizing dry surfaces. UV-C, which has a shorter wavelength, has a higher light energy level than UV-B and UV-A; therefore, it is widely used for water or liquid food sterilization [19, 20]. In recent years, there has been an increased need for purifying drinking water using household filter water purifiers [21]. Cartridge filter water purification systems are typically combined with other treatment devices, such as UV disinfection systems [3]. The technologies of household water purifier devices with cartridge filtration and UV disinfection, which are readily available in the market, are widely used globally [22]. Nevertheless, there is a lack of scientific literature on the commercial systems based on these technologies and evaluating their effectiveness. However, *Shigella* spp. and *Listeria monocytogenes* have been known as representative major waterborne and foodborne pathogens worldwide and their contamination in drinking water could cause waterborne and foodborne illness [23, 24]. So, the purpose of this study was to investigate the microbial disinfection effect and physiological characteristics of the Gram-negative bacterium *S. flexneri* and Gram-positive bacterium *L. monocytogenes*using a household water purifier with a wavelength in the UV-C region.

## Materials and Methods

### Bacterial Strains

*Shigella flexneri* ATCC 12022, a Gram-negative bacterium and *Listeria monocytogenes* ATCC 19115, a Gram-positive bacterium were used in this study. A colony of each bacterial strain was inoculated into Tryptic Soy Broth (TSB, Cat. No. 211825, BD, USA) or Brain Heart Infusion broth (BHI, Cat. No. 237500, BD, USA) and cultured at 37°C with vigorous shaking. An overnight culture of each bacterial strain was used for the water purification experiments. For the selective media of *S. flexneri* ATCC 12022 and *L. monocytogenes* ATCC 19115, *Salmonella*-Shigella agar (SSA, Cat. No. 274500, USA) and modified Oxford medium agar (Cat. No. 222530, BD, USA; Modified Oxford Antimicrobial Supplementation, CatD17, 2163, BD, USA) were used.

### Water Purification System with UV-C and Carbon Filter

The water purifier (eSpring, Amway Co. Ltd., USA) and UV-carbon filter were provided by Amway Korea (Amway Korea Ltd., Korea). For the UV-treated samples, the carbon filter was removed from the UV-carbon filter and only a UV lamp was used for the experiment. For the carbon filter samples, only the UV-carbon filter was used for the experiment, while the UV lamp was turned off. The UV lamp was a mercury lamp with a wavelength of 254 nm, an irradiation dose of 40 mJ/cm^2^, a diameter of 12 mm, and a height of 195 mm, with two tubes connected. The UV lamp was covered with a quartz tube (30 mm in diameter, 200 mm in height), a stainless steel tube (50 mm in diameter, 200 mm in height) was installed on the outside of the quartz tube, and water was passed from the bottom to the top. The area irradiated with UV was the size of a stainless steel tube, the passage time of water was 4.4 s, and UV was irradiated only when water flowed. The UV water purification system was designed to be sealed, except for the inlet and outlet pipes, by vertically combining the UV lamp inside the cylinder with a capacity of 2.8 L. The flow rate of the water purifier was adjusted to 3.4 L/min using a diaphragm pump (22R-3005, KOTEC, Korea), and measured using a flow meter (FLM-3, HM Digital Inc., USA). Bacteria were injected into the water purifier at a rate of 9 ml/min using a syringe pump (NE-300, New Era Pump Systems Inc. USA).

### Determination of Microbial Growth on Water Purifier with Carbon Filter, UV-C and UV-C/Carbon Filter

The bacterial culture in the liquid medium was transferred to a microtube and centrifuged (10,000 ×*g*, 1 min; Labogene, Korea) to harvest the bacteria, after which, the supernatant was removed. To remove the medium completely, the bacterial pellet was resuspended in 1 ml of 0.85% NaCl solution and centrifuged (10,000 ×*g*, 1 min) to collect the bacteria. The washing step was repeated twice, post which, the collected bacterial strain was resuspended in 0.85 NaCl and adjusted to an optical density (O.D.) A_600nm_ = 0.5 using a UV-visible spectrophotometer (Optizen 2120UV, K LAB Co., Ltd., Korea). The prepared bacterial solution was serially diluted to concentrations of 10^6^, 10^7^, and 10^8^ CFU/ml in 0.85% NaCl solution and was introduced (injected) into the water purifier with 4 L of drinking water at a flow rate of 3.4 L/min. Drinking water (Jeju Samdasoo, Kwang Dong Pharmaceutical Co., Ltd., Seoul, South Korea) used in this study was purchased from a local market. Drinking water samples containing bacteria were treated with a carbon filter or UV-C irradiation, or both carbon filter and UV-C irradiation. The drinking water sample was passed through a water purifier collected in a glass bottle, and bacterial strains were retrieved using the membrane filtration method (Pall Co., USA) with a membrane disk filter (PES membrane, pore size: 0.2 μm, size 47 mm, Pall Co.) and a vacuum instrument. For selective culture medium, the membrane disc filter containing retrieved bacteria was placed in close contact with the surface of each selected medium (Salmonella-Shigella agar or modified Oxford medium agar) and incubated at 37°C for 24 h. Colony forming units (CFU) were calculated to confirm the growth inhibition ability of the water purifier under the conditions of carbon filter, UV-C irradiation, and UV-C irradiation combined with carbon filter. For fluorescence microscopy and flow cytometry, the membrane disc filter was placed in a 50 ml conical tube containing 5 ml of 0.85% NaCl solution and vortexed vigorously for 5 min to separate the bacteria from the membrane filter.

### Time-Coursed Determination of Bacteria After UV-C Irradiation

The artificially inoculated bacteria with a concentration of 10^8^ CFU/ml were irradiated with UV-C through a water purifier and were then collected on a membrane disc filter through membrane filtration. The membrane disc filter was transferred to a 50 ml conical tube, 5 ml of 0.85% NaCl solution was added, and the tube was vortexed for 5 min. The bacterial suspension was transferred to a 1.7 ml microtube and centrifuged (10,000 ×*g*, 1 min), and the supernatant was discarded and resuspended in 0.85% NaCl solution. The collected resuspended bacterial samples were stored for 0, 4, 8, 12, and 24 h after UV-C treatment in the laboratory at 25°C and used for fluorescence microscopy and flow cytometry.

### Florescence Microscope and Flow Cytometry Measurements

The bacterial samples collected and separated from the membrane filter were stained using the LIVE/DEAD BacLight bacterial viability kit (L-13152, Invitrogen/Thermo Fisher Scientific, USA) according to the manufacturer`s instructions. In brief, equal volume of staining solution was added to the sample, stored for 15 min in the dark for staining, centrifuged (10,000 ×*g*, 1 min), the supernatant was removed, and washed twice with 1 ml of 0.85% NaCl solution. The pellet was resuspended in 100 μl 0.85% NaCl solution. To use the live bacterial control sample as an indicator, freshly cultured bacteria washed twice was used. For the dead bacterial control sample, bacteria were placed in 70% isopropanol (300 μl) for 1 h and washed twice with 0.85% NaCl solution and centrifuged. The live/dead stained bacterial samples were analyzed using a fluorescent microscope (KB-2000F, Korea Lab Tech, Korea) and flow cytometry (CytoFlex, Beckman Coulter, USA) to observe the morphological and flow cytometric characteristics of bacteria in the water purifier with and without UV-C irradiation.

## Results

### Disinfection Effects of Water Purifier with Carbon Filter and UV-C Irradiation

Before the water purification experiments, correlations between O.D. (A_600nm_) vs CFU were estimated (data not shown). Bacterial culture (O.D. A_600nm_ = 0.5) was serially diluted 10-fold and spread on TSA and SSA for *S. flexneri*, and on BHI agar and Oxford agar for *L. monocytogenes*. *L. monocytogenes* ATCC 19115 grown on BHI agar (~5.2 × 10^8^ CFU/ml) was similar to that on Oxford agar (~5.0 × 10^8^ CFU/ml). However, *S. flexneri* ATCC 12022 showed different CFUs of around 1.6 × 10^8^ CFU/ml on TSA and 0.8 × 10^8^ CFU/ml on SSA. These different CFU results based on the media suggest that SSA may be stringent for some strains of *Shigella* spp., especially when the culture is stressed [25, 26].

**Fig. 1:**
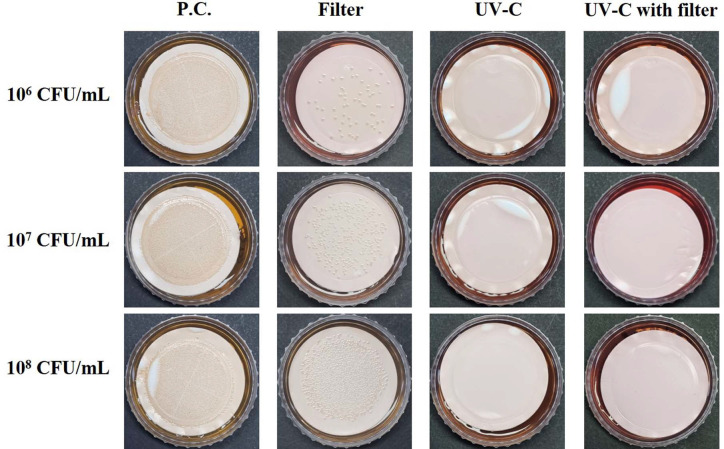
Growth of *Shigella flexneri* in water samples, which were artificially *S. flexneri* inoculated and passed through the water purifier, on *Salmonella*-Shigella agar. Positive control (P.C.): no carbon filter, no UV-C irradiation; filter: with carbon filter; UV-C: with UV-C irradiation; UV-C with filter: with carbon filter and UV-C irradiation.

**Fig. 2:**
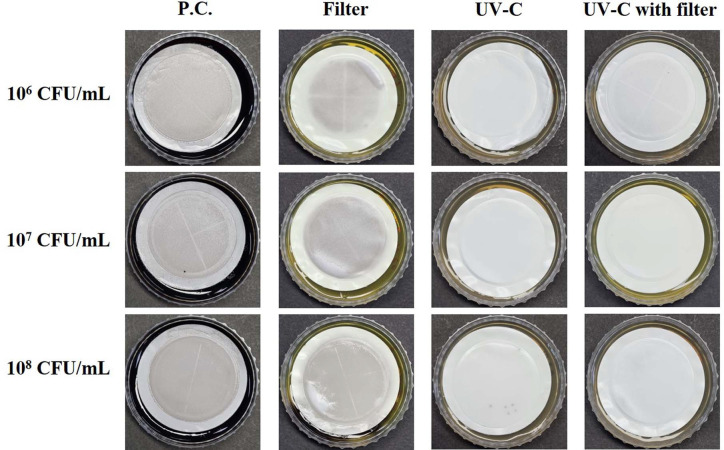
Growth of *Listeria monocytogenes* in water samples, which were artificially *L. monocytogenes* inoculated and passed through the water purifier, on Oxford agar. Positive control (P.C.): no carbon filter, no UV-C irradiation; filter: with carbon filter; UV-C: with UV-C irradiation; UV-C with filter: with carbon filter and UV-C irradiation.

The disinfection and reduction effects of the water purifier were evaluated at three bacterial concentrations (10^6^, 10^7^, and 10^8^ CFU/4 L) under three conditions: carbon filter, UV-C irradiation, and UV-C irradiation with carbon filter. The results of the membrane filtration method are shown in Figs. 1 and 2. *S. flexneri* (Gram-negative bacterium) was trapped on the filter disc and overgrew (too numerous to count, TNTC) on SSA as a positive control sample (Fig. 1). The carbon filter of the water purifier showed reduction effect of *S. flexneri* to some extent, but at high concentrations, the filtration yield of *S. flexneri* was decreased (Fig. 1, Filter, 10^6^ CFU/4 L: 63 CFUs; 10^7^ CFU/4 L: ~ 290 CFUs; 10^8^ CFU/4 L sample: TNTC). However, UV-C irradiation in water purifiers showed strong disinfection effects on *S. flexneri* (Fig. 1, UV-C and UV-C with filter, no colony formed) even at high concentrations. For *L. monocytogenes* (Gram-positive bacterium) (Fig. 2, UV-C and UV-C with filter), UV-C irradiation also showed high disinfection effects at high concentrations, but the Oxford agar in the 10^8^ CFU sample with UV-C irradiation (Fig. 2, UV-C, 10^8^ CFU/4 L sample; UV-C with filter, 10^8^ CFU/4 L sample, medium color of the reverse side) turned from bright yellow to black, which implied the presence of surviving *L. monocytogenes* (Oxford agar is a selective medium for *L. monocytogenes*, identified as black colonies), whereas the carbon filter of the water purifier showed little effect on the reduction of *L. monocytogenes* (Fig. 2 Filter, TNTC with smeared dark-gray colonies). These results imply that UV-C irradiation of the water purifier has a growth inhibition effect or a high disinfection effect on both *S. flexneri* and *L. monocytogenes*.

### Flow Cytometric Features with UV-C Irradiation

**Fig. 3:**
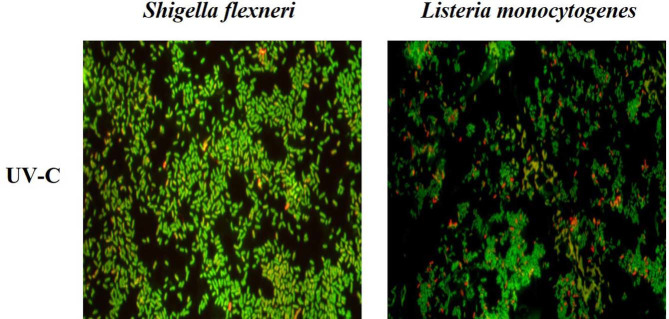
Fluorescence microscope images of *S. flexneri* and *L. monocytogenes* treated with UV-C irradiation through the water purifier.

**Fig. 4:**
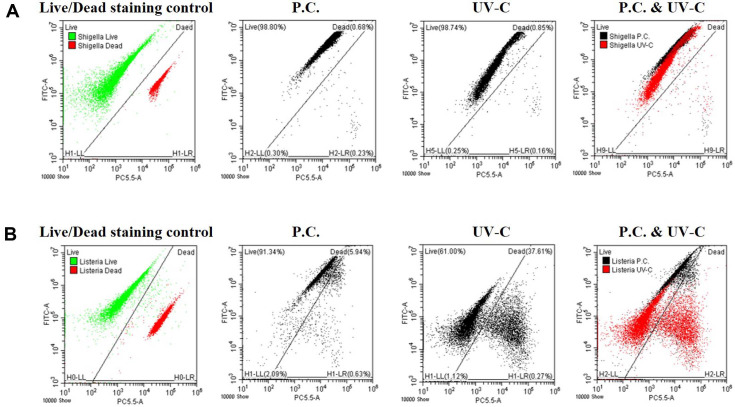
Flow cytometric analysis of *S. flexneri* (A) and *L. monocytogenes* (B) treated with/without UV-C irradiation through the water purifier. Live/dead staining control: live bacteria (green color), dead bacteria (red color); positive control (P.C.): retrieved bacteria without UV-C irradiation; UV-C: retrieved bacteria with UV-C irradiation; P.C. & UV-C: overlapped picture of P.C. and UV-C; Axis FITC-A: green fluorescence, Axis PC5.5-A: red fluorescence.

To determine the effect of UV-C irradiation on the morphological and physiological shift of bacteria or on the degree of bacterial live/death, *S. flexneri* and *L. monocytogenes* were subjected to fluorescence microscopy and flow cytometry using the LIVE/DEAD BacLight bacterial viability kit immediately after UV-C irradiation through the water purifier, as shown in Figs. 3 and 4. Live and dead controls for *S. flexneri* and *L. monocytogenes* were prepared. Live bacteria showed green fluorescence with SYTO 9 reagent and dead bacteria (or bacteria with damaged cell walls) showed red fluorescence with propidium iodide reagent, which penetrates only bacteria with damaged cell membranes, in the live/dead staining kit under fluorescence microscopy (data not shown). Monitoring of UV-C-irradiated bacteria through fluorescence microscopy showed that *S. flexneri* (Gram-negative bacteria) showed a green and yellow color tendency; however, *L. monocytogenes* (Gram-positive bacteria) showed a higher frequency of red color than *S. flexneri* (Fig. 3).

Flow cytometric monitoring of UV-C-irradiated bacteria revealed notable results for *S. flexneri* and *L. monocytogenes*. The positive control of *S. flexneri* (Fig. 4A, P.C.) showed high green fluorescence, which slightly decreased after UV-C irradiation (Fig. 4A, UV-C). However, P.C. of *L. monocytogenes* (Fig. 4B, P.C.) showed similar flow cytometry results as *S. flexneri*, but interestingly, UV-C-irradiated *L. monocytogenes* (Fig. 4B, UV-C) shifted to reduced green fluorescence and divided into two groups with red fluorescence intensity, which showed completely different plot patterns compared with *S. flexneri*. Forward scatter (FSC, relative cell size) and side scatter (SSC, relative cell granularity) were measured between the P.C. and UV-C samples for both bacteria, and there were no significant differences in morphological characteristics (data not shown). These results imply that the shifts in fluorescence from green (P.C.) to red (UV-C) originated from differences in the degree of staining with live/dead staining reagents, not from explosion or other physical variations of the cell wall after UV-C irradiation.

### Time-Coursed Monitoring on Fluorescence Microscopy and Flow Cytometry after UV-C Irradiation

**Fig. 5:**
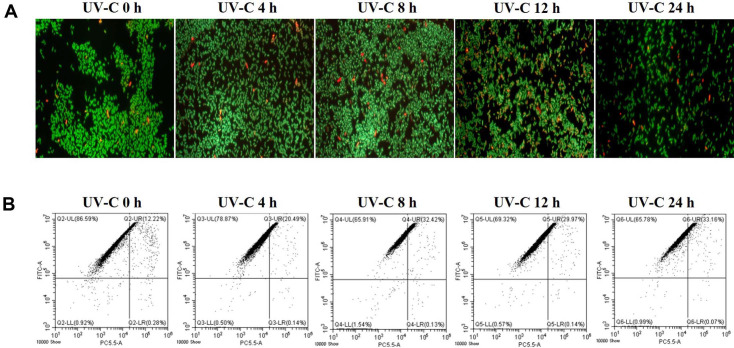
Time-course analysis of *S. flexneri* after UV-C irradiation through the water purifier using fluorescence microcopy (A) and flow cytometry (B). Axis FITC-A: green fluorescence, Axis PC5.5-A: red fluorescence.

**Fig. 6:**
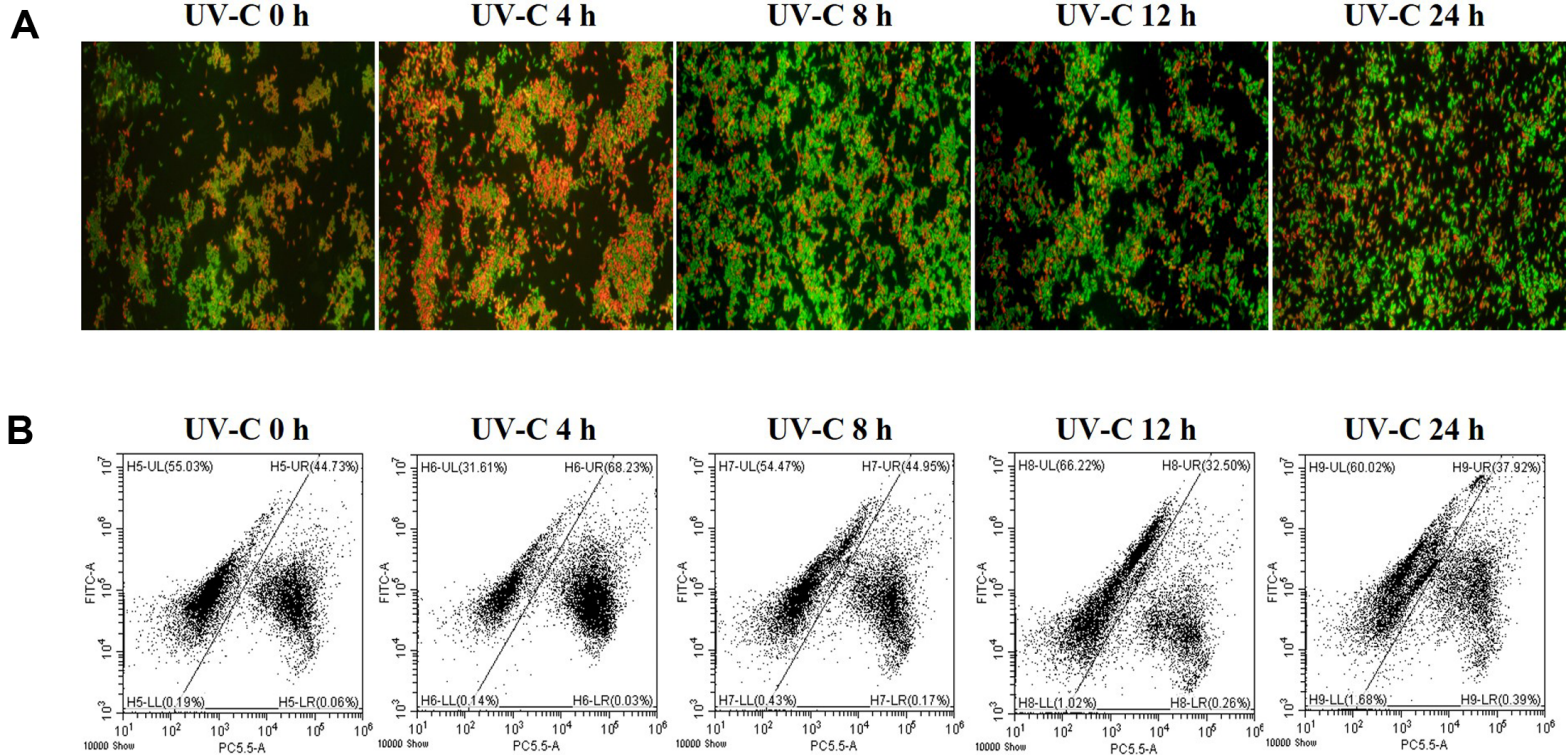
Time-course analysis of *L. monocytogenes* after UV-C irradiation through the water purifier using fluorescence microcopy (A) and flow cytometry (B). Axis FITC-A: green fluorescence, Axis PC5.5-A: red fluorescence.

UV-C-irradiated *S. flexneri* and *L. monocytogenes* (10^8^ CFU/4 L) were monitored from 0 to 24 h using fluorescence microscopy and flow cytometry to measure bacterial characteristics over time and to understand the effects of UV-C irradiation (Figs. 5 and 6). In *S. flexneri*, the red fluorescence slightly and gradually increased from 0 to 24 h on axis PC5.5-A in the dot plot (Fig. 5B), whereas the green fluorescence was maintained throughout the flow cytometric analysis, which was identical to the fluorescence microscopy results as strong green fluorescence (Figs. 5A and 5B). Interestingly, in *L. monocytogenes*, the strong red fluorescence dots (dead) on axis PC5.5-A were at a high proportion (around 40%) at 0 h, increased up to 68% at 4 h, and then decreased to mid-30–40% from 8 h to 24 h in the flow cytometric analysis (Fig. 6B). These results were identical to the fluorescence microscopy images (Fig. 6A). These results provide basic data on the physiological changes in Gram-positive and Gram-negative bacteria over time following UV-C irradiation.

## Discussion

Among the non-thermal treatment techniques, UV-C irradiation has great potential as a sterilization method for microorganisms in food matrices. Therefore, studies on the disinfection and sterilization of pathogenic microorganisms in food or drinking water using UV-C irradiation have been reported to promote food storage and ensure food safety [9, 27-29]. The sterilization mechanism of UV-C on bacteria can be explained as follows: Nikogosyan and Görne [30] reported that UV radiation acts on organic substances, especially hydrocarbons, to cause changes in their physical and chemical structures. The double helix structure of DNA is lost by acting on the components of microorganisms, particularly cytosine and thymine [16, 17]. A representative mechanism of bacterial resistance to UV-C irradiation is photoreactivation, which reverses DNA lesions by photolyase enzymes using the energy of visible light [31-33]. It is also known that resistance to UV is generally higher in Gram-positive bacteria, due to their DNA repair ability and thick peptidoglycan layer, than in Gram-negative bacteria [18, 28, 34-37]. Our results for the membrane disc filter on selective medium (Figs. 1 and 2) showed that UV-C-irradiated *S. flexneri* perished at low and high concentrations (10^6^–10^8^ CFU/4 L water), but UV-C-irradiated *L. monocytogenes* survived at high concentrations (10^8^ CFU/4 L water) by forming colonies. These results are consistent with previous reports that Gram-positive bacteria have relatively higher resistance to UV-C irradiation. Notably, fluorescence microscopy and flow cytometric analysis combined with live/dead staining showed different characteristics between *S. flexneri* and *L. monocytogenes* after UV-C irradiation. Immediately after UV-C irradiation, most individual cells maintained green fluorescence (or yellowish green) under fluorescence microscopy for both bacteria, which might indicate their survival (Fig. 3). However, flow cytometric analysis provided unexpected results in *L. monocytogenes* (Gram-positive bacteria), which were divided into two groups by high and low red fluorescence intensity with drastically decreased green fluorescence, whereas *S. flexneri* (Gram-negative bacteria) still maintained high or slightly decreased green fluorescence (Fig. 4). The reason for this drastic shift of *L. monocytogenes* in dot plots of the flow cytometric analysis remains unknown and might have been caused by heavy alteration of lipid and protein compositions in the cell membrane structure or destruction of DNA structure affecting permeability/binding strength of fluorescence dye in *L. monocytogenes* [35, 38-40].

Time-course monitoring provided more significant results. In *S. flexneri*, it was difficult to confirm the change in fluorescence over time after UV-C irradiation using a fluorescence microscope. However, flow cytometry observations confirmed that the intensity of red fluorescence slowly and gradually increased over time (Fig. 5), suggesting that UV-C irradiation led *S. flexneri* to be inactivated over time. On the contrary in *L. monocytogenes*, fluorescence microscopy and flow cytometry suggested that cell membranes were temporarily damaged immediately after UV-C irradiation (Fig. 6; 0 and 4 h, increased red fluorescence group, up to 68%) and gradually recovered over time (Fig. 6; 8, 12, and 24 h, red fluorescence decreased). These results suggest that responses of *L. monocytogenes* to UV-C irradiation are more immediate than those of *S. flexneri* and might be consistent with previous reports that Gram-positive bacteria have superior resistance to UV-C irradiation compared with Gram-negative bacteria [18, 28, 34-37].

Overall, the results of our study imply that bacteria do not die immediately after UV-C irradiation, but under the influence of UV-C irradiation, physiological functions are disrupted, growth ability is lost, and the bacteria are gradually inactivated. However, although a small number of *L. monocytogenes* survived at high concentrations under UV-C and UV-C with carbon filter conditions (Fig. 2), UV-C irradiation in the water purifier showed sufficiently high inactivation and reduction effects on both Gram-positive and Gram-negative bacteria, considering the high concentration of bacteria used in this study, which is hardly seen in daily life.

This study investigated the inactivation and flow cytometric characteristics of *S. flexneri* and *L. monocytogenes* in drinking water using UV-C irradiation in a water purifier system. UV-C irradiation (wavelength of 254 nm and 40 mJ/cm^2^) in the water purifier exhibited sufficient inactivation effects from low to high concentration (*S. flexneri*, 1.6 × 10^8^ CFU/4 L; *L. monocytogenes*, 5.2 × 10^8^ CFU/4 L) on both bacteria. Flow cytometry and fluorescence microscopic observation with live/dead staining indicated that *S. flexneri* seemed to be weakened to the death phase over time after UV-C irradiation, whereas *L. monocytogenes* seemed to be the weakest at 4 h after UV-C irradiation, followed by a recovery pattern. Adding a UV-C sterilizer to a water purifier may be effective for microbial safety of water, and the results of this study can provide basic data for the use of the UV sterilization system, aiding in future research.
